# Automated mobile virtual reality cognitive behavior therapy for aviophobia in a natural setting: a randomized controlled trial

**DOI:** 10.1017/S0033291722003531

**Published:** 2023-10

**Authors:** T. Donker, J.R. Fehribach, C. van Klaveren, I. Cornelisz, M. B. J. Toffolo, A. van Straten, J.-L. van Gelder

**Affiliations:** 1 Department of Clinical, Neuro and Developmental Psychology, Vrije Universiteit Amsterdam and Amsterdam Public Health Research Institute Amsterdam, Amsterdam, Noord-Holland, The Netherlands; 2 Department of Biological Psychology, Clinical Psychology and Psychotherapy, Albert Ludwigs-University of Freiburg, Freiburg im Breisgau, Baden-Württenberg, Germany; 3 Department of Education Sciences, Section Methods and Statistics, Vrije Universiteit Amsterdam, Amsterdam, Noord-Holland, The Netherlands; 4 Amsterdam Center for Learning Analytics (ACLA), Amsterdam, Noord-Holland, The Netherlands; 5 Department of Criminology, Max Planck Institute for the Study of Crime, Security and Law, Freiburg im Breisgau, Baden-Württenberg, Germany; 6 Department of Clinical Neurodevelopmental Sciences, Institute of Education and Child Studies, Leiden University, Leiden, Zuid-Holland, The Netherlands

**Keywords:** Automated, aviophobia, smartphone application, specific phobia, virtual reality

## Abstract

**Background:**

Access to evidence-based psychological treatment is a challenge worldwide. We assessed the effectiveness of a fully automated aviophobia smartphone app treatment delivered in combination with a $5 virtual reality (VR) viewer.

**Methods:**

In total, 153 participants from the Dutch general population with aviophobia symptoms and smartphone access were randomized in a single-blind randomized controlled trial to either an automated VR cognitive behavior therapy (VR-CBT) app treatment condition (*n* = 77) or a wait-list control condition (*n* = 76). The VR-CBT app was delivered over a 6-week period in the participants' natural environment. Online self-report assessments were completed at baseline, post-treatment, at 3-month and at 12-month follow-up. The primary outcome measure was the Flight Anxiety Situations Questionnaire (FAS). Analyses were based on intent-to-treat.

**Results:**

A significant reduction of aviophobia symptoms at post-test for the VR-CBT app compared with the control condition [*p* < 0.001; *d* = 0. 98 (95% CI 0.65–1.32)] was demonstrated. The dropout rate was 21%. Results were maintained at 3-month follow-up [within-group *d* = 1.14 (95% CI 0.46–1.81)] and at 12-month follow-up [within-group *d* = 1.12 (95% CI 0.46–1.79)]. Six participants reported adverse effects of cyber sickness symptoms.

**Conclusions:**

This study is the first to show that fully automated mobile VR-CBT therapy delivered in a natural setting can maintain long-term effectiveness in reducing aviophobia symptoms. In doing so, it offers an accessible and scalable evidence-based treatment solution that can be applied globally at a fraction of the cost of current treatment alternatives.

## Introduction

It is estimated that one in 10 people worldwide require mental health care at any one point in time, yet only nine mental health workers are available for every 10 000 people globally (World Health Organization, 2018). If anything, the coronavirus disease 2019 (COVID-19) pandemic has further exacerbated this already challenging state of affairs as the prevalence of mental health problems has increased dramatically (Winkler et al., 2020), while access to evidence-based treatment has declined (World Health Organization, 2020).

With the post-pandemic reopening of societies, novel challenges relating to ‘re-entry anxiety’ (American Psychological Association, 2021) have appeared. Re-entry anxiety, or spontaneous recovery, refers to the re-emergence of conditioned responding to an extinguished conditioned stimulus (Craske, Treanor, Conway, Zbozinek, & Vervliet, 2014). Specifically, the lack of exposure to feared objects or situations (e.g. social interactions, flying) has resulted in increased anxiety levels in phobics and an overall increase in the prevalence of phobias (American Psychological Association, 2021; Winkler et al., 2020). In addition, phobias may increase the risk of developing other anxiety disorders (Trumpf, Margraf, Vriends, Meyer, & Becker, 2010) and depression (Choy, Fyer, & Goodwin, 2007). Hence, the need for scalable solutions to meet these challenges is acute. In this study, we test the effectiveness of a low-cost, scalable, and standalone virtual reality exposure therapy (VRET) for one of the most prevalent phobias, namely aviophobia (Curtis, Magee, Eaton, Wittchen, & Kessler, 1998).

VRET has shown effectiveness in reducing aviophobia symptoms (Cardoş, David, & David, 2017; Fodor et al., 2018; Krijn et al., 2007; Rothbaum et al., 2006). For example, Rothbaum et al. (2006) were one of the first to compare VRET to standard *in vivo* treatment and a waitlist control condition in a randomized controlled trial (RCT) among 75 participants and concluded that VR exposure was superior to waitlist, and essentially equivalent to exposure *in vivo*. Yet, despite promising effects, clinical virtual reality (VR) has faced implementation challenges due to high costs, technical obstacles, and lack of availability (Geraets, Van der Stouwe, Pot-Kolder, & Veling, 2021). The development of consumer VR platforms has led to the emergence of automated VR treatment, with encouraging results (Bentz et al., 2021; Freeman et al., 2018; Miloff et al., 2019). Generally, specialized equipment remains required, however, and prior efforts have all involved contact with a research team or therapist, thus limiting their scalability. One recent study, however, demonstrated that automated VR-CBT for treating acrophobia in a natural setting, without human contact is feasible and effective, also at 3-month follow-up (Donker et al., 2019). In addition, the reduction in acrophobia symptoms was larger when the feeling of being present in the VR and user-friendliness were higher (Donker et al., 2019).

To demonstrate the potential of fully automated mobile virtual reality cognitive behavior therapy (VR-CBT) for more complex disorders, and also its long-term potential, we examined the effectiveness of a VR-CBT app for aviophobia. Aviophobia is a heterogeneous condition with large variation in onset and acquisition characteristics and with high rates of comorbidity with other phobias and anxiety disorders. This makes aviophobia more difficult to treat than other phobias (Van Almen & Van Gerwen, 2013). To ensure scalability, our treatment relies on low-cost and widely available equipment – a participant's own smartphone and a $5 cardboard VR viewer – and was delivered in a natural setting without human contact.

We hypothesized that the VR-CBT app would demonstrate a greater reduction in flight anxiety, general anxiety, and depressive symptoms from pre- to post-treatment compared to the wait-list control condition, and that these effects would be maintained at 3-month and 12-month follow-up. Furthermore, we hypothesized that the app would be rated as user-friendly and satisfactory, and that a higher sense of presence in VR, higher perceived realism of VR, and higher user-friendliness would all be associated with a larger reduction in fear-of-flying symptoms at post-test.

## Method

### Participants and design

Participants for this two-arm, single-blind RCT were recruited from the Dutch general population through the placement of advertisements on websites and local media. Data were collected pre-treatment, post-treatment, and at 3-month and 12-month follow-up. Ethics approval was granted by the Medical Ethical Committee of the Vrije Universiteit Medical Center (registration number: 2019-321). The study was prespecified in the trial protocol (Fehribach et al., 2021). No changes to methods or trial outcomes were made after trial commencement and no data were analyzed before study completion. Results are published in accordance with CONSORT guidelines for RCTs (Schulz, Altman, & Moher, 2010). Eligible for inclusion were individuals who scored ⩾ 56 (indicating mild flight anxiety) on the Flight Anxiety Situations Questionnaire (FAS) (Van Gerwen, Spinhoven, Van Dyck, & Diekstra, 1999), had smartphone access (⩾ iPhone 5, or Android with ⩾ Lollipop 5.1 OS, gyroscope, and 4.7–5.5 inches screen size), were between the ages of 18 and 64, and provided written informed consent. Individuals who were currently being treated for any phobia, were taking psychotropic medication (unless dosage had been stable for ⩾ 3 months and no changes were planned during study period), and/or had insufficient Dutch language proficiency were excluded from participation. Participants provided written informed consent after receiving a complete description of the study. This study is registered in the Netherlands Trial Registry (NL 70238.029.19., https://www.trialregister.nl/trial/8257). All procedures contributing to this work comply with the ethical standards of the relevant national and institutional committees on human experimentation and with the Helsinki Declaration of 1975, as revised in 2008.

### Randomization and masking

A randomization list using Sealed Envelope™ Random Allocation Software (https://www.sealedenvelope.com/simple-randomiser/v1/lists) was created and kept by an independent researcher who revealed the next randomization outcome after every inclusion, thus ensuring that the research team was blind to treatment allocation. Block randomization (using blocks of 6, 8, 10, and 12 participants) with 1:1 allocation was used. The allocation sequence was concealed from the researchers. Randomization was performed after pre-treatment assessment. Participants were aware of which arm they had been allocated to. All self-report measures were completed online.

### Intervention: VR-CBT app

The treatment was delivered through a VR-CBT app that participants could download on their own smartphone in combination with a $5 VR viewer. The VR-CBT app was launched as a beta test in both the Google Play store (Android) and the App store (IOS), meaning that the app could only be accessed through an URL and a unique pin code for each participant that was provided by the research team. Technical questions from participants were answered by e-mail by a research team member.

The VR-CBT app consisted of six CBT-based animated modules that included a virtual therapist (modeled on the first author). The modules took between 5 and 40 min each to complete. The app also included severalgamified immersive VR environments and interactions such as checking in at an airport, the boarding process, finding one's seat, taking off, flying with turbulence, and landing (see online Supplementary Fig. SF1 and a video SV1 in the online supplement). The scenarios covered the entire exposure spectrum of flight anxiety and was developed based on input from a user-test among 15 participants suffering from flight anxiety. Examples of (gamified) interactions included locating objects that passengers brought onto the airplane (scavenger hunt) and comforting another nervous passenger. In addition, the app included sounds typical of airplane travel (e.g. engine roar, announcements from the cabin crew, chattering voices). Participants were able to progress to subsequent exposure levels if they rated their anxiety as ⩽3 on a ten-point scale after finishing a level. Participants who rated the VR level with a score of >3 received a message explaining that they should practice the same level again before moving on to the next level. Virtual environment navigation occurred via gaze control. See Fehribach et al. (2021) for intervention details. The scenarios were developed by the first and last author (TD, JLvG) in collaboration with app developers and designers. The VR environment was developed with the Unity game engine (version 2018.4.1f1; Unity Technologies). Participants followed the therapy in their own time and natural environment and were encouraged to complete it within 6 weeks.

The VR-CBT app is available in Google Play (for Android devices) and the App Store (for Apple smartphones) for about $10. The app runs on nearly all smartphones from 2016 or newer (as long as they are equipped with a gyroscope). In terms of hardware, only a rudimentary VR viewer is required (cost approx. $5–10).

## Outcomes

### Primary outcome measure

#### Flight Anxiety Situations Questionnaire

The 32-item Flight Anxiety Situations Questionnaire (FAS) (Van Gerwen et al., 1999) was used to assess flight anxiety (item score 1–5; total score range 32–160) at base-line, post-test and follow-up. The FAS is reliable and has excellent validity with a cut-off score of 56 (Nousi, Van Gerwen, & Spinhoven, 2008). The Cronbach Alpha in this study was 0.97.

### Secondary outcome measures

#### Flight Anxiety Modality Questionnaire

The 18-item Flight Anxiety Modality Questionnaire (FAM; Van Gerwen et al., 1999) targets flight anxiety as well (item score 1–5; total score range 18–90) and was included to test for the robustness of the results. The reliability is good (Nousi et al., 2008). The FAM was assessed at all time-points.

### Beck Anxiety Inventory

The Beck Anxiety Inventory (BAI; Beck, Epstein, Brown, and Steer, 1988), Dutch translation (Beck & Steer, 2015) consists of 21 items (item score 0–4, total score range 0 to 63) measuring general anxiety and has good reliability and validity (Brown, Beck, Newman, Beck, & Tran, 1997). The BAI was assessed at all time-points.

### Patient health questionnaire

The 9-item Patient Health Questionnaire (PHQ-9) (Kroenke, Spitzer, & Williams, 2001), Dutch version was used to assess depressive symptoms at all time-points. Validity is good (Wittkampf, Naeije, Schene, Huyser, & van Weert, 2007).

### Web screening questionnaire

Three items from the baseline Web Screening Questionnaire (Donker, Van Straten, Marks, & Cuijpers, 2009) [items pertaining to panic disorder, agoraphobia, and obsessive-compulsive disorder (OCD)], were assessed to examine whether they influenced post-test flight anxiety outcomes. Reliability and validity of these items was found to be good (Donker et al., 2009).

### Credibility/Expectancy questionnaire

The 6-item Credibility/Expectancy Questionnaire (CEQ; Devilly and Borkovec, 2000), Dutch version (Mertens, Moser, Verbunt, Smeets, & Goossens, 2017) was used to asses self-reported treatment expectations at baseline (intervention group only). Reliability and validity of CEQ was found to be good (Devilly & Borkovec, 2000; Mertens et al., 2017).

### Client satisfaction questionnaire

The Dutch version of the 8-item Client Satisfaction Questionnaire (CSQ-8; Attkisson & Greenfield, 2004; de Brey, 1983) assessed global client treatment satisfaction at post-test (intervention group only) using scale response options from 1–4 (total score range: 8–32) and has found to be reliable and valid (de Brey, 1983).

### System usability scale

The 10-item System Usability Scale (SUS; Bangor, Kortum, and Miller, 2008) measured user-friendliness of the VR-CBT app at post-test [item score range 0–5, total score was calculated and converted to range 0–100 (44)] with scores of at least 70 are considered as passable and better products ranging into the 80s. Reliability and validity are good (Bangor et al., 2008). This measure was administered to the intervention group only.

### Igroup presence questionnaire

The 14-item Dutch version of the Igroup Presence Questionnaire (IPQ; (Schubert, Friedmann, & Regenbrecht, 2001) assessed realism and the feeling of ‘presence’ or immersion in the VR environments (item score range −3 to 3; total score range −42–42). The IPQ demonstrated reliability ((Schubert et al., 2001) and was administered at post-test to the intervention group only.

### Additional questions

Professional aviophobia treatment usage, assessed at each time point, and vision and hearing impairment were included as control variables. Adverse events were inquired about at post-test and follow-up.

All assessments were self-report measures completed online in participants' natural environment and outside researchers' presence and were programmed with Survalyzer software (http://www.survalyzer.com).

## Statistical analyses

A full statistical analysis plan was designed before the trial (Fehribach et al., 2021). Power calculations were based on the FAS, the primary outcome measure. A previous meta-analysis that examined the effect of VRET on fear-of-flying symptoms (Fodor et al., 2018) yielded an effect size of Cohen's *d* = 0.82. However, because the VR-CBT app is self-guided and uses rudimentary equipment, a more conservative estimate of Cohen's *d* = 0.70 was used. We determined that 34 participants per group would provide 80% power at a 5% significance level (two-tailed). With an anticipated dropout rate of 40% (see Donker et al., 2019), 114 participants were required (*n* = 57 per condition).

Baseline characteristics were tabulated for each condition. Differences across the two groups were assessed with chi-square tests or analyses of variance, as appropriate. To assess whether dropout cases (participants who discontinued treatment after commencing) and complete cases (participants who completed post-test) were non-random, we constructed a balancing table comparing the background characteristics, pre-scores, and other covariates of participants with and without missing outcome observations. Missing outcome values for the dropout sample were imputed using a multiple imputation (MI) procedure that exploited pre-scores and a set of pre-specified background characteristics (gender, age, severity of symptoms, and a dummy variable for whether a participant had missing values on one or more of the background characteristics). Ordinary least squares regression models with pre-scores and background characteristics included were used on an intention-to-treat (ITT) basis to estimate the treatment effect. In addition to standardized mean differences (Cohen's *d*), we conducted two robustness analyses. First, because non-random sample attrition may bias the estimated treatment effects (Cornelisz, Cuijpers, Donker, & Van Klaveren, 2020; Donker et al., 2019), nonparametric treatment effect bounds were estimated using the Random Forest Lee Bounds (RFLB) procedure (Lee, 2009; Schonlau & Zou, 2020). Second, MI using an iterative Markov chain Monte Carlo (MCMC) method (Hamra, MacLehose, & Richardson, 2013) based on initial treatment assignment was conducted to deduce the statistical significance of the reported differences between conditions. Furthermore, potential heterogeneity and mechanisms of treatment were analyzed by estimating the variation in treatment effects with pre-scores, general anxiety, user-friendliness, treatment expectation/credibility and satisfaction, and presence. To investigate whether fear-of-flying symptom severity at baseline predicted outcome, we analyzed the predicted reductions in post-test score for 10-point bins of pre-scores using regression analysis. A sensitivity analysis was conducted by examining whether the results among complete cases (participants who completed post-test and/or follow-ups) differed from the results of the ITT analysis. Clinically meaningful changes on the FAS were analyzed for the ITT sample using the ‘method (C)’ clinically significant change (CSC) formula described by Evans (Evans, 1998) with an FAS score of *M* = 41.55 (s.d.: 11.64) (Skolnick, Schare, Wyatt, & Tillman, 2012). The reliable change criterion was computed based on the standard deviation of the FAS pre-test scores and the pre-test Cronbach's alpha of the FAS (*α* = 0.97) (ITT sample). This yielded a standard error of change of 4.67 and, in turn, a reliable change criterion of 9.15 (4.67 × 1.96) points on the FAS (Christensen & Mendoza, 1986; Jacobson & Truax, 1991). Finally, using the formula by Furukawa and Leucht (2011), the number-needed-to-treat (NNT) was assessed for both the ITT sample and the complete-case sample based on the size of the effects observed on the FAS. For all analyses, two-sided *p* values < 0.05 were taken to indicate statistical significance. Stata (version 16; StataCorp) was used for the analyses.

## Results

### Participants

Enrollment commenced on 9 November 2019 and ceased on 7 May 2020. Data analysis began in December 2020.

**Fig. 1.**
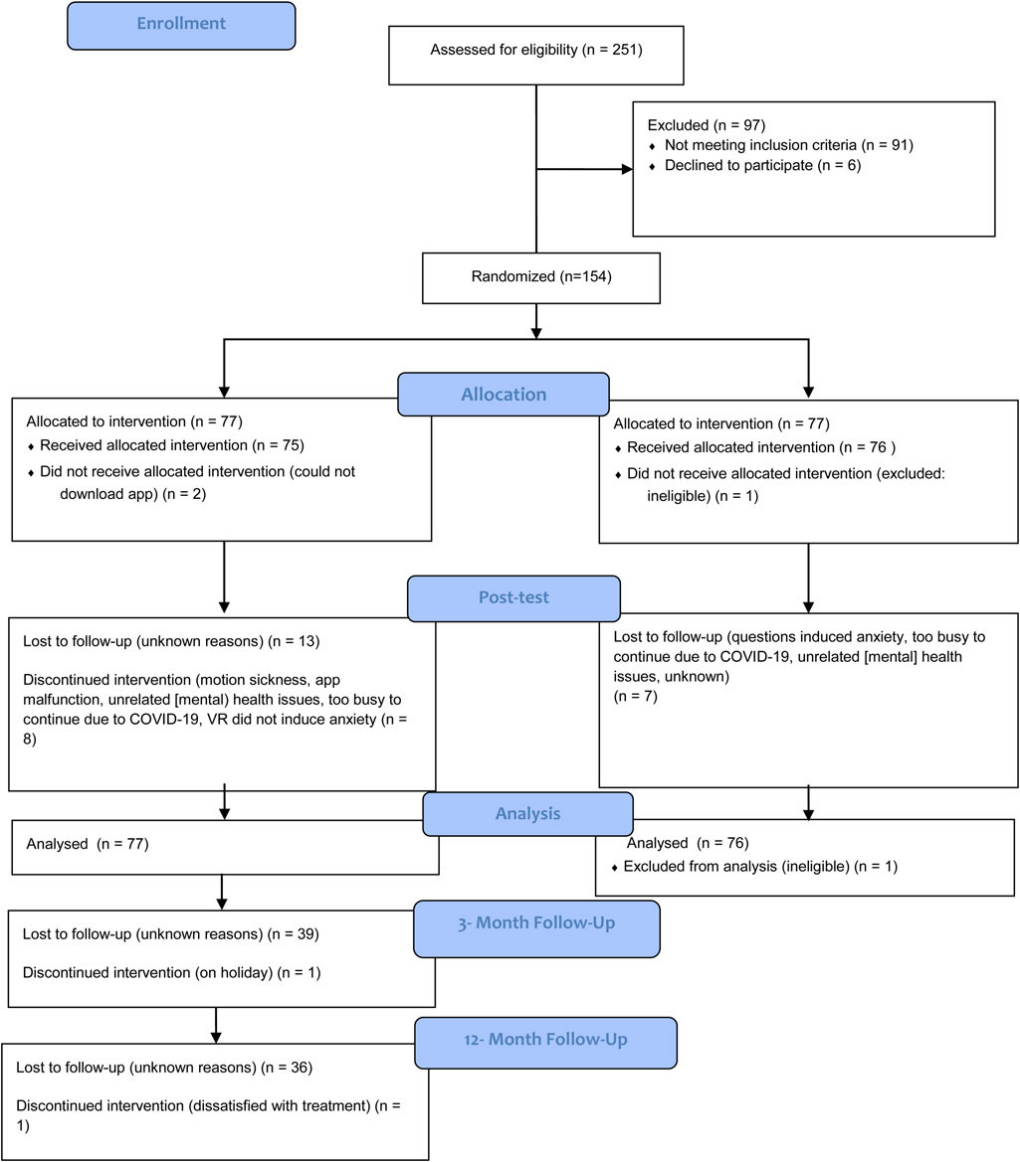
CONSORT diagram of participant flow through the trial. Abbreviations: FAS, Flight Anxiety Situations Questionnaire; VR, virtual reality.

Between 9 November 2019 and 7 May 2020, 251 individuals self-referred online for the study, of whom 204 proceeded to screening. Of these, 50 were ineligible based on the exclusion criteria. Accordingly, between 15 November 2019 and 7 May 2020, 154 participants were randomized to the VR-CBT app treatment condition (*n* = 77) or the wait-list control condition (*n* = 77). However, due to a technical error, one participant in the control condition was excluded after randomization, but before treatment commencement, because the participant scored below the cut-off on the baseline FAS. The last participant completed 12-month follow-up data on 20 July 2021. See Fig. 1 for participant flowchart.

Participants in the two conditions were balanced with regard to the baseline characteristics and demographic variables (Table 1). Most participants had experienced fear-of-flying symptoms for longer than 5 years (121 of 153, 79%). Sixteen participants (10%) had no flight experience whatsoever.

**Table 1.** Demographic and baseline characteristics^a^

	VR treatment group (*n* = 77)		Wait-list control group (*n* = 76)		
Measure	Mean	s.d.	Mean	*SD*	p-value diff
Age (years)	41	12.50	43	11.79	0.314
	*n*	%	*n*	%	
Female	64	83.11	63	82.89	0.696
Education					
Primary	1	1.30	1	1.32	0.993
Secondary	9	11.69	12	15.79	0.464
Postsecondary	67	87.01	63	15.79	0.479
Employment status					
Full-time employed	36	46.75	32	42.11	0.566
Part-time employed	35	45.45	37	48.68	0.691
Unemployed	6	7.79	7	9.21	0.755
Marital status					
Single	10	12.99	8	10.53	0.639
Married, living together	56	72.73	49	64.47	0.274
Other	11	14.29	19	25.00	0.096
Psychotropic medication	7	9.09	4	5.26	0.363
Duration of fear of flying					
< 6 months	0	0.00	0.00	0.00	n/a
6–12 months	2	2.60	2	2.63	0.990
1–5 years	13	16.88	15	19.74	0.651
> 5 years	62	80.52	59	77.63	0.663
Never flown in an airplane	10	12.99	6	7.89	0.307
Smartphone type					
iPhone	37	48.05	40	52.63	0.574
Android	40	51.95	36	47.37	0.574

a Abbreviations: VR, virtual reality.

### Treatment adherence and attrition

Of the 77 participants in the intervention group, 54 (70%) completed the post-test, 37 (49%) completed the 3-month follow-up, and 40 (52%) completed the 12-month follow-up; in the wait-list group, 91% (69 of 76) completed the post-test. Dropout (32 of 153, 21%) was not related to background characteristics, pre-scores, or other covariates except for educational level. Specifically, a higher proportion of participants who completed only secondary education did not drop out (*z* = 2.44, *p* = 0.015). Also, a higher proportion of participants who completed postsecondary education *did* drop out (*z* = −2.00, *p* = 0.046) (online Supplementary Table ST1 in the online supplement). Therefore, outcome values were not missing completely at random. This was addressed by using RFLB and MI.

### Effectiveness: Intention-to-treat analysis

Compared to the control condition, the VR-CBT app treatment condition exhibited a significant reduction in fear-of-flying symptoms as measured by the primary outcome measure (FAS) at post-test [*b* = −21.90 (95% CI −28.65 to −15.14), *t*_147_ = −6.44, *p* < 0.0001]. The effect size was *d* = 0.98 (95% CI 0.65–1.32). The NNT was 3.3. In addition, a significant difference at post-test between the intervention and the control group was also observed on the secondary fear-of-flying outcome measure (FAM), [*b* = −12.68 (95% CI −18.13 to −7.23), *t*_147_ = −4.62, *p* *<* 0.0001; *d* = 0.62 (95% CI 0.29–0.94)). No differences were observed for general anxiety (BAI) [*b* = −2.02 (95% CI −6.48–2.45), *t*_147_ = −0.90, *p* = 0.372; *d* = 0.09 (95% CI −0.23 to 0.40)] or depressive symptoms (PHQ-9) [*b* = −0.68 (95% CI −1.82 to 0.45), *t*_147_ = −1.20, *p* = 0.234; *d* = 0.25 (95% CI −0.07 to 0.57)]. Full results of the ITT analysis are shown in Tables 2 and 3.

**Table 2.** Outcome measure scores, intention to treat (*n* = 153)^a^

	VR treatment group (*n* = 77)		Wait-list control group (*n* = 76)	
Outcomes	Mean	s.d.	Mean	s.d.
*Primary outcome*				
FAS				
Baseline	106.66	18.32	104.32	19.83
Post-test	83.09	19.29	103.71	22.58
*Secondary outcome*				
FAM				
Baseline	69.25	15.80	68.04	17.92
Post-test	69.25	17.51	69.63	18.58
BAI				
Baseline	12.96	13.51	11.59	12.05
Post-test	13.56	13.11	14.74	13.93
PHQ				
Baseline	3.82	4.60	4.45	4.44
Post-test	3.39	3.34	4.30	3.99

Abbreviations: VR, virtual reality; FAS, Flight Anxiety Situations Questionnaire; FAM, Flight Anxiety Modality Questionnaire; BAI, Beck Anxiety Inventory; PHQ, Patient Health Questionnaire.

a Inferential statistics of treatment outcome measures, single-regression-based imputation.

**Table 3.** Estimated effects of VR-CBT app on fear of flying, general anxiety, and depression^a^

Post-test^b^								
	Model 1 (*n* = 153)				Model 2 (*n* = 121)		Model 3 (*n* = 153)	
Measure	*b* ITT (s.e.)	Adj. *R*	*p* value^c^	Effect size (95% CI)	*b* Lower bound (s.e.)	*b* Upper bound (s.e.)	*b* MI (s.e.)	*p* value^c^
Primary outcome								
FAS	−22.55 (2.41)	0.61	<0.0001	0.98 (0.65–1.32)	−31.59 (4.46)	−14.25 (4.76)	−21.90 (3.40)	<0.0001
Secondary outcomes								
FAM	−12.38 (2.04)	0.57	<0.0001	0.62 (0.29–0.94)	−19.90 (3.97)	−4.93 (4.05)	−12.68 (2.75)	<0.0001
BAI	−2.16 (1.63)	0.46	0.190	0.09 (−0.23 to 0.40)	−5.67 (3.24)	5.22 (2.64)	−2.02 (2.25)	0.372
PHQ	−0.54 (0.41)	0.55	0.194	0.25 (−0.07 to 0.57)	−2.21 (0.86)	0.88 (0.66)	−0.68 (0.57)	0.234

a Abbreviations: VR, virtual reality; ITT, intent-to-treat; FAS, Flight Anxiety Situations Questionnaire; FAM, Flight Anxiety Modality Questionnaire; BAI, Beck Anxiety Inventory; PHQ, Patient Health Questionnaire; MI, multiple imputation, s.e., standard error.

b For ethical reasons, we do not have follow-up data for the wait-list control group participants: they were given access to the VR treatment after the post-test. Hence, we could not analyze the follow-up results for the intent-to-treat sample.

c Two-sided. Treatment effects reported in the preferred, conservative model (Model 1) are ITT effects based on single-regression-based imputation using initial assignment status, pre-score, gender, age, and a dummy variable for whether a participant had missing values on one or more background characteristics. Treatment effects reported in Model 2 are Lee Bounds estimates. Treatment effects in Model 3 are derived through multiple (50) imputations, assuming missing at random, applying multivariate normal regression with imputation by experimental group, and accommodating arbitrary missing outcome value patterns using an iterative MCMC method. Pre-score and background controls are included for improved precision of the regression point estimates reported in Models 1 and 3. Background control variables are gender, age, and a dummy variable for whether a participant had missing values on one or more background characteristics. Effect sizes reported in Models 1 and 3 are all Cohen's *d* for unadjusted group means based on the pre-measure–post-measure difference.

### Robustness and sensitivity analyses

Because there was a differential dropout rate between the two conditions, RFLB were estimated (see Lee, 2009; Schonlau and Zou, 2020). The resulting upper and lower bounds (see Table 3, Model 2) confirmed the statistically significant treatment effect on the primary outcome measure (FAS). The regression coefficient estimated in Model 1 (*b* = −22.55, s.e. = 2.41) lies in the middle of the Lee Bounds.

Because one participant reported at post-test that s/he had received professional aviophobia treatment during the time between baseline and post-test, a robustness analysis was conducted by repeating the ITT analysis with this participant excluded. This did not influence the results significantly [FAS: *b* = −21.96 (95% CI −28.78 to −15.14), *t*_146_ = −6.39, *p* *<* 0.0001]. Nine participants indicated that they had received professional aviophobia treatment during the time between 3-month and 12-month follow-up. A robustness analysis excluding these participants indicated that this did not influence the primary outcome results significantly (*p* = 0.70); the within-group effect size of the 31 participants that did not receive professional aviophobia treatment between follow-ups was *d* = 1.06 (95% CI 0.53–1.60). As indicated by the robustness analysis, the VR-CBT app impacted the anxiety specific to aviophobia, and general anxiety did not drive the results (BAI: *b* = −2.02, s.e. = 2.25, *p* = 0.37).

### Heterogeneous treatment effects and mechanisms

**Fig. 2.**
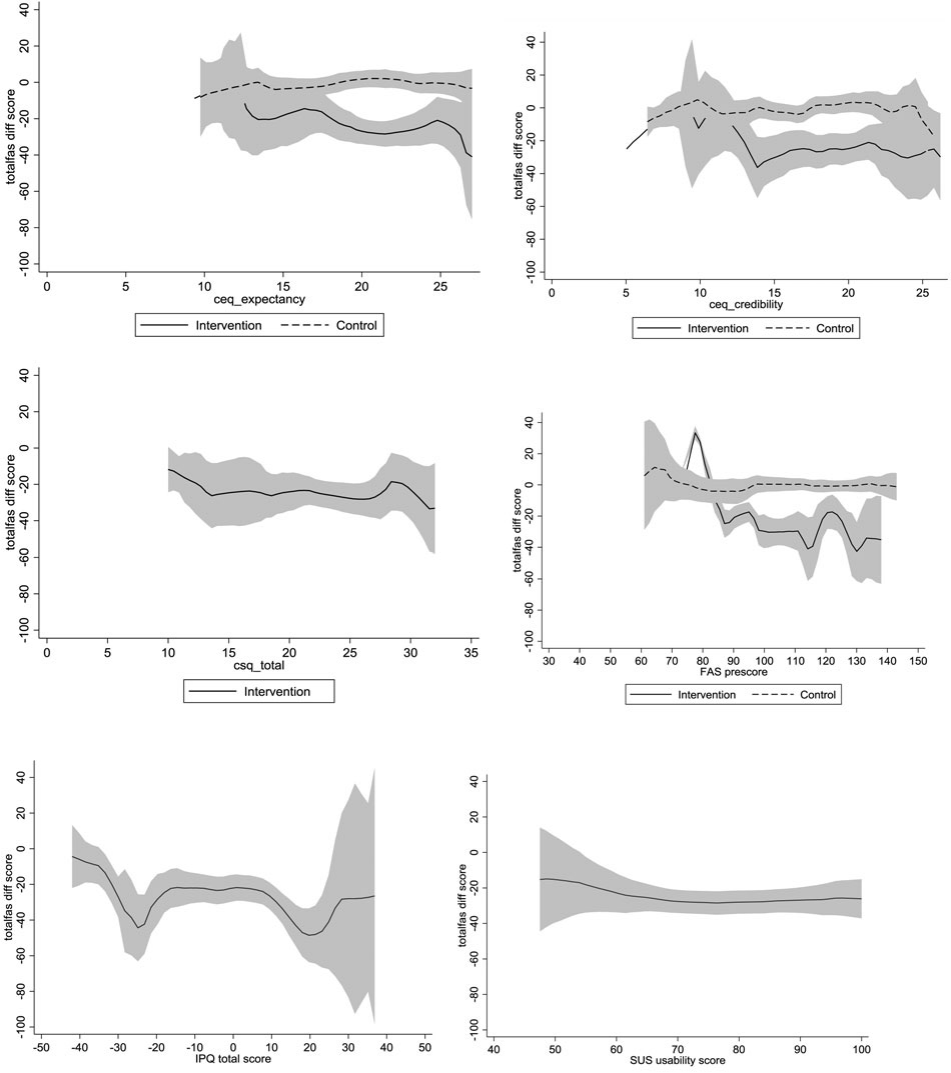
Heterogeneous treatment effects and mechanisms. The graphs show the change in FAS difference scores by the VR-CBT app CEQ expectancy (*n* = 54), CEQ credibility (*n* = 54), CSQ satisfaction (*n* = 54), FAS pre-score (*n* = 54), IPQ (*n* = 54), and SUS usability score (*n* = 53), from baseline to 6 weeks' post-test. Values reflect least-square means; error bars indicate standard error of the mean. Abbreviations: CEQ, Credibility Expectancy Scale; CSQ, Client Satisfaction Scale; FAS, Flight Anxiety Situations Questionnaire; IPQ, Igroup Presence Questionnaire; SUS, System Usability Scale.

Baseline aviophobia symptoms influenced treatment effectiveness: participants with more severe fear-of-flying symptoms at baseline benefitted more from the VR-CBT app (*b* = −0.0579, s.e. = 0.173, *p* *<* 0.0001). Participants who thought that the app would be less credible before the start of the treatment (CEQ–credibility) derived less benefit than those who rated the app as more credible (Fig. 2). However, no significant differences in post-treatment FAS scores were found between those scoring high and those scoring low on treatment expectancy (CEQ–expectancy), usability of the VR-CBT app (SUS), satisfaction with the VR-CBT app (CSQ), or presence (IPQ) (Fig. 2). Likewise, having a visual impairment was not related to post-treatment FAS scores (*b* = −0.94, s.e. = 6.05, *p* = 0.89). We also explored whether underlying symptoms of panic disorder (*n* = 84), OCD (*n* = 88), and claustrophobia (*n* = 81) – that is, symptoms likely to influence participants' flight anxiety – affected the results. With little statistical power, no statistically significant differences in treatment effectiveness were observed for these subgroups of participants.

### Complete cases

The between-group FAS effect size for those who completed post-test was *d* = 0.98 (95% CI 0.60–1.36). The NNT was 3.3. In total, 41 of 54 participants (76%) demonstrated reliable change. In addition, among participants who completed post-test, 14 of 54 (26%) experienced CSC (a FAS change of ⩾ 65.80). At 3-month and 12-month follow-up, the within-group FAS effect size increased to *d* = 1.14 (95% CI 0.46–1.81) and *d* = 1.12 (95% CI 0.46–1.79) respectively. A medium effect size at 3-month and 12-month follow-up was obtained for the FAM (*d* = 0.78 and *d* = 0.67 respectively), but no significant within-group change between baseline and follow-ups was observed for general anxiety and depression (online Supplementary Table ST2 in the online supplement). The total mean IPQ score at post-test (intervention group only) was −1.30 (s.d. = 17.22, observed range: −42–42). The results on flight usage were almost certainly impacted by the COVID-19 pandemic, during which recruitment took place and during which air travel was restricted and therefore not shown.

### User-friendliness, treatment satisfaction, and adverse effects

The VR-CBT app was rated as user-friendly (SUS: *M* = 79.38, s.d. = 13.78). Treatment expectation and credibility (CEQ) before commencing VR-CBT treatment was *M* = 36.94 (s.d. = 7.19; range: 14.6–53.2). Total mean treatment satisfaction (CSQ) was 22.17 (s.d. = 4.62; range: 10–32). No deterioration or negative effects as defined by Rozental et al. (2018) were identified except for six participants who reported one or more symptoms of transient cyber sickness and two participants who had VR viewer problems (i.e. missing strap).

## Discussion

The results of this single-blind RCT demonstrated that a VR-CBT app using a rudimentary cardboard VR viewer and delivered in a natural setting without human contact was effective in reducing fear-of-flying symptoms at post-test and at both 3-month and 12-month follow-up. The effect sizes are similar to therapist-guided *in vivo* exposure for specific phobias (Wolitzky-Taylor, Horowitz, Powers, & Telch, 2008). Importantly, our results are also within the range of what is reported in meta-analyses of therapist-guided, high-end VR studies targeting fear of flying (Cardoş et al., 2017; Fodor et al., 2018). Furthermore, the app was rated as both user-friendly and satisfactory by participants, and dropout rates were similar to those reported in therapist-guided VR studies (Benbow & Anderson, 2019). No significant changes emerged for VR-CBT participants on general anxiety and depression. This is likely due to the low baseline levels of general anxiety and depression. Surprisingly, even though participants reported relatively low levels of presence, this was unrelated to treatment effectiveness. Although this finding was contrary to expectations, previous research has demonstrated mixed findings regarding the influence of presence on effectiveness (e.g. Donker et al., 2019; Fodor et al., 2018; Miloff et al., 2019; Price & Anderson, 2007). The influence of presence on treatment outcomes requires more research in order to better understand how it interacts with anxiety and treatment outcome, and to clarify the causal relationship between presence and fear (Botella, Fernández-Álvarez, Guillén, García-Palacios, & Baños, 2017). Interestingly, whereas those who rated the VR-CBT app as less credible pre-treatment, also derived less benefit from the VR-CBT app. Post-treatment satisfaction rates were unrelated to effectiveness. Moreover, treatment expectancy, user-friendliness, and underlying causes of aviophobia (e.g. panic disorder) did not influence responses on the primary outcome measure. However, power was low for these exploratory analyses, which could explain the absence of significant findings. Notably, only 26% of participants completing the app reached CSC, meaning that the majority of the participants still suffered from some degree of flight anxiety. This could be related to the complex and heterogeneous nature of the condition compared to other phobias, which makes it more difficult to treat (Van Almen & Van Gerwen, 2013). However, as the large effect size of the primary outcome measure demonstrates, participants who used the VR-CBT app derived significant benefit from it.

It has been argued that VR exposure lowers the threshold for exposure *in vivo* (Lindner et al., 2021). This study was carried out during the COVID-19 Pandemic. It is therefore likely that the effects of the current intervention would have been even larger if participants were able to take actual flights. In addition, because of its portability, the VR-CBT app can easily be used to practice with exposure between flights.

One of the main strengths of this trial was that the intervention took place in the participants' natural environment, thus increasing ecological validity. Furthermore, any influence of human contact on the findings was ruled out because participants did not receive any therapist guidance during treatment or when responding to assessments. Additional strengths were that the treatment had cross-platform compatibility (i.e. could be used on both iPhone and Android smartphones) and that the treatment was developed for common smartphones (up to approximately 5 years old), thereby increasing generalizability. Finally, this is the first study to demonstrate that the effects of fully automated VR-CBT in a real-life setting can be maintained long term.

This study also had several limitations. First, the Simulator Sickness Questionnaire, which is commonly used to measure cyber sickness, could not be included because it overlaps with symptoms of anxiety and might therefore not be a valid measure of cyber sickness when used in studies of phobia treatment (Donker et al., 2019). Consequently, we were unable to assess participants' cyber sickness levels during the trial. We could therefore not establish whether low presence levels reported in this study might have been caused by cyber sickness. However, only six participants reported mild symptoms of cyber sickness at post-test. Second, our data relied entirely on self-report measures. However, previous research comparing self-report and behavioral measures revealed no differences between them (Morina, Ijntema, Meyerbröker, & Emmelkamp, 2015). Finally, the research design allowed for assessing the effectiveness of the therapy as a whole but not for assessing specific components, as our sample size precluded the possibility of examining change mechanisms. Future research is needed to examine which treatment components influence the effectiveness of the treatment, and to examine possible predictors or moderators of treatment effectiveness (e.g. presence, cyber sickness, education level) and drop out in automated VR research.

## Conclusions

Mental healthcare access has traditionally been a global challenge. If anything, the COVID-19 pandemic has accelerated the need for accessible, scalable, and affordable evidence-based mental health care, especially now that, as society reopens, ‘re-entry anxiety’ is on the rise. The results of this study confirm that a low-cost, highly scalable, and fully automated VR-CBT app can be an accessible and effective health care solution for reducing phobia symptoms. In doing so, it offers an accessible and scalable evidence-based treatment solution that can be applied globally at a fraction of the cost of current treatment alternatives.

## Supplementary material

For supplementary material accompanying this paper visit https://doi.org/10.1017/S0033291722003531. click here to view supplementary material
