# Supplementary material for: Automated mobile virtual reality cognitive behavior therapy for aviophobia in a natural setting: a randomized controlled trial
Source: Psychol Med. 2022 Nov 25;53(13):6232–41. doi: 10.1017/S0033291722003531 (PMC10520596; doi:10.1017/S0033291722003531)
Supplement: Supplementary file 1 [file S0033291722003531sup.zip › S0033291722003531sup002.docx]

**Supplementary online content**

Donker et al: Automated mobile virtual reality cognitive behavior therapy for aviophobia in a natural setting: A randomized clinical trial.

**Figure SF1.** Screenshots from the VR-CBT app and VR environment

**Table ST1.** Comparison of missing‑outcome participants to non-missing‑outcome participants after randomization

**Table ST2.** Inferential statistics of treatment outcome measures, complete cases (*n*= 121–123)

**Video SV1.** Video of the VR environment.

**Figure SF1. Screenshots from the VR-CBT app and VR environment**

**
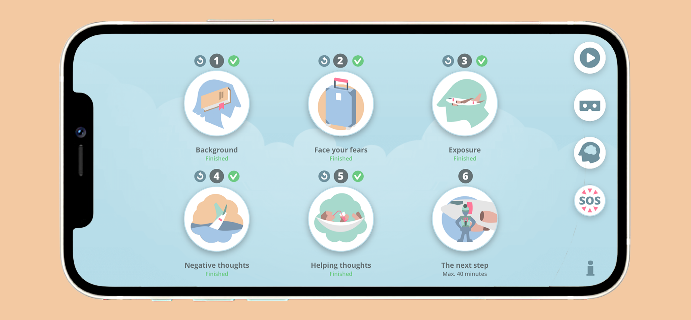

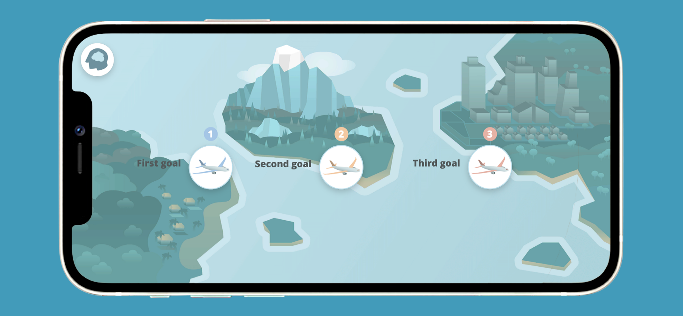


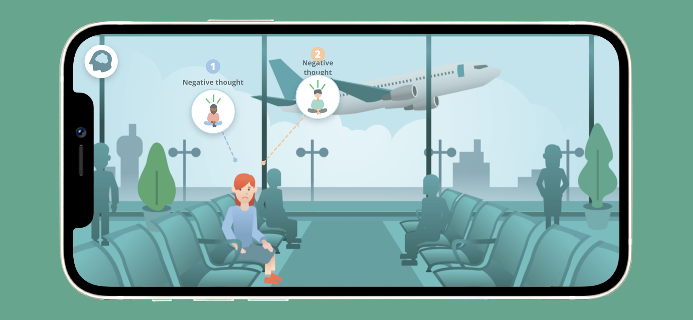

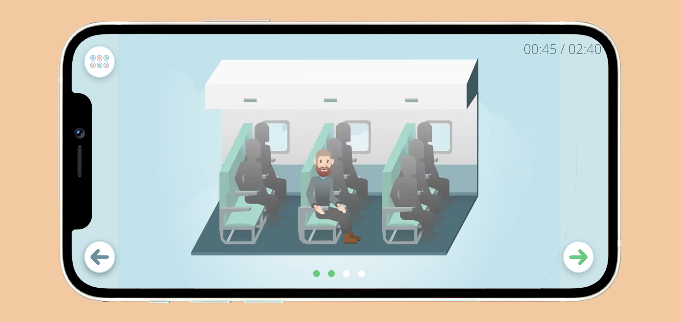
**


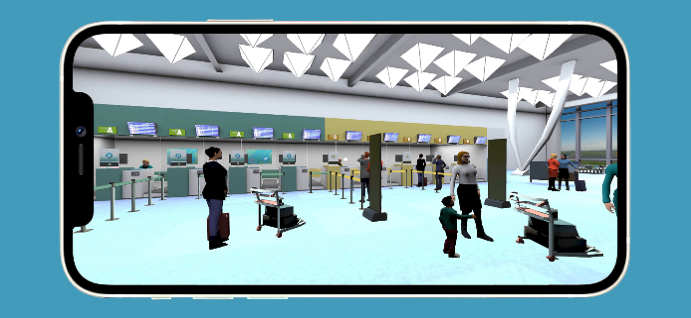

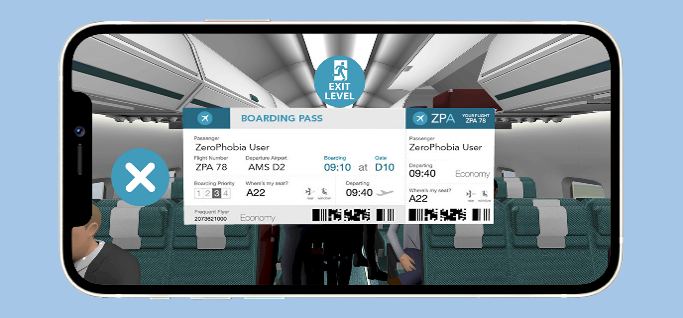


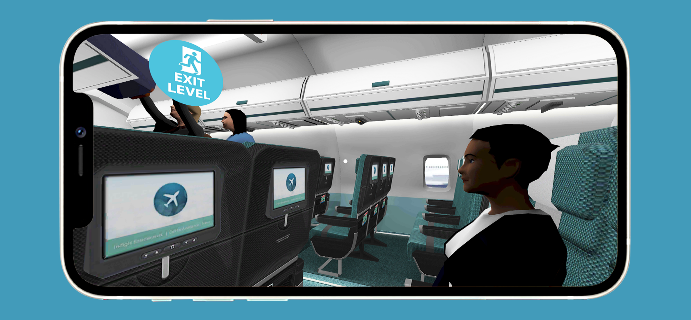


**Table ST1. Comparison of missing‑outcome participants to non-missing‑outcome participants after randomization^a^**

|  | Non-missing (*n* = 123) | | Missing (*n* = 30) | | *p* value |
| --- | --- | --- | --- | --- | --- |
|  | **Mean** | ***SD*** | **Mean** | ***SD*** |  |
| Demographic variables |  |  |  |  |  |
| Age | 42.34 | 12.49 | 39.73 | 10.56 | 0.29 |
|  | ***n*** | **%** | ***n*** | **%** |  |
| Female | 105 | 85.37 | 22 | 73.33 | 0.12 |
| Education |  |  |  |  |  |
| Primary | 1 | 0.81 | 1 | 3.33 | 0.28 |
| Secondary | 21 | 17.07 | 0 | 0.00 | 0.015 |
| Postsecondary | 101 | 82.11 | 29 | 96.67 | 0.046 |
| Psychotropic medication | 8 | 6.50 | 3 | 10.00 | 0.51 |
| Primary outcome - baseline |  |  |  |  |  |
|  | **Mean** | ***SD*** | **Mean** | ***SD*** |  |
| FAS | 105.34 | 18.67 | 106.13 | 20.89 | 0.84 |
| Secondary outcome - baseline |  |  |  |  |  |
| FAM | 68.32 | 16.61 | 70.00 | 18.01 | 0.63 |
| BAI | 13.50 | 10.44 | 17.41 | 15.00 | 0.24 |
| PHQ | 4.32 | 3.59 | 5.62 | 4.81 | 0.18 |

^a^ Abbreviations: FAS = Flight Anxiety Situations Questionnaire; FAM = Flight Anxiety Modality Questionnaire; BAI = Beck Anxiety Inventory; PHQ = Patient Health Questionnaire.

**Table ST2. Inferential statistics of treatment outcome measures, complete cases (*n*= 121–123)^a^**

|  | **VR treatment group**  **(*n*= 40–54)** | | | | **Wait‑list control group (*n* = 68–69)** | | ***p* value^b^** | **Effect size (Cohen’s *d*) (95% CI)** | | |
| --- | --- | --- | --- | --- | --- | --- | --- | --- | --- | --- |
| **Primary outcome (*M*, *SD*)** | Baseline | Post-test | 3-month FU | 12-month FU | Baseline | Post-test |  | Between, post-test | Within, Baseline-3-month FU ^c^ | Within, Baseline-12-month FU ^c^ |
| FAS | 106.11 (17.95) | 82.46 (20.71) | 78.36 (22.22) | 83.28 (21.35) | 104.74 (19.32) | 104.06 (22.86) | <0.0001 | 0.98  (0.61–1.36) | 1.14  (0.46–1.81) | 1.12  (0.46–1.79) |
| **Secondary outcomes (*M*, *SD*)** |  | | | | | | | | | |
| FAM | 68.09 (14.73) | 69.91 (19.03) | 52.62 (17.50) | 57.13 (16.55) | 68.49 (18.04) | 69.91 (19.03) | <0.0001 | 0.66  (0.29–1.02) | 0.78  (0.31–1.25) | 0.70  (0.06–1.34) |
| BAI | 12.00 (11.72) | 13.06 (13.53) | 8.22 (6.90) | 10.40 (10.27) | 11.42 (11.87) | 14.59 (14.49) | 0.554 | 0.11  (-0.25–0.47) | 0.35  (-0.10–0.81) | 0.09  (-0.53– .71) |
| PHQ | 3.39 (3.96) | 3.17 (3.36) | 3.76 (3.20) | 3.43 (3.71) | 4.28 (4.46) | 4.18 (4.14) | 0.152 | 0.26  (-0.10–0.62) | 0.04  (-0.42–0.49) | -0.05  (-0.67– -0.57) |
|  | | | | | | | | | | |
|  | | | | | | | | | | |

^a^ Abbreviations: VR = Virtual Reality; FAS = Flight Anxiety Situations Questionnaire; FAM = Flight Anxiety Modality Questionnaire; BAI = Beck Anxiety Inventory; PHQ = Patient Health Questionnaire; FU = follow-up.
^b^ *t*-test comparisons of baseline and post-test scores for complete cases (two-tailed).
^c^ Within-group effect sizes are based on participants who responded at both baseline and the applicable follow-up time point
